# Supplementary material for: CT perfusion stroke lesion threshold calibration between deconvolution algorithms
Source: Sci Rep. 2023 Dec 5;13:21458. doi: 10.1038/s41598-023-48700-6 (PMC10698076; doi:10.1038/s41598-023-48700-6)
Supplement: Supplementary file 1 — Supplementary Information. [file 41598_2023_48700_MOESM1_ESM.docx]

**Supplemental Materials**

*Generating the Digital Perfusion Phantom*

A digital perfusion phantom uses Equation (1) to simulate tissue TDCs from an assumed arterial TDC and simulated IRFs with known ground truth parameters. The quantitative accuracy of a deconvolution algorithm can then be benchmarked by deconvolving the arterial TDC from the simulated TDC to estimate perfusion parameters and compare against the known ground truth values.

In our digital perfusion phantom, the arterial TDC was taken from the internal carotid artery in the healthy brain hemisphere of an acute ischemic stroke CT perfusion (CTP) study with a uniform image interval of 1.8 s over 81 s. No further filtering or curve fitting was applied. Flow-scaled IRFs were simulated as gamma-variate functions to ensure that they had a different shape than the IRFs used in the model-based deconvolution methods. The gamma-variate IRF was written in the form:

$$R^{GV}\left( t \right)=\left\{ \begin{aligned} 0, &\tau<0 \\ \tau^{\alpha}e^{\alpha\left( 1-\tau\right)}, &\tau\geq0 \end{aligned} \right.$$

where $\tau=(t-T_{0})/T_{max}$, $\alpha$ is an exponential scaling parameter used to set the $MTT$, and $T_{max}$ is the peak time of the gamma-variate function. $T_{max}$ was fixed to $T_{0}+0.5MTT$ to reduce the number of free parameters. For a prescribed set of $T_{0}$ and $MTT$ values, $\alpha$ was computed numerically by determining the $\alpha$ required for $R^{GV}\left( t \right)$ to have an area equal to the $MTT$.

Gamma-variate IRFs were simulated with a wide range of ground truth perfusion parameters: $T_{0}\in[0.0, 0.5, 1.0, 2.0, 3.0, 4.0, 8.0]$ s, $MTT\in[3.4, 4.0, 6.0, 8.0, 10.0, 12.0, 16.0]$ s, and $CBV\in[0.5, 1.0, 1.5, 2.0, 2.5, 3.0, 4.0, 5.0]$ ml/100 g. CBF was calculated as CBV/MTT by the Central Volume Principle;^18^ accordingly, CBF ranged from 1.9 to 88.2 ml/min/100 g at non-uniform intervals. Ground truth tissue TDCs were calculated by numerically convolving the simulated IRF and the linearly interpolated patient arterial curve at 0.01 s interval then resampled at 2 s interval. Zero-mean Gaussian noise with standard deviation, $\sigma=1.5$ HU was added to the tissue TDCs to simulate the expected noise variation in tissue TDCs after standard Gaussian filtering of dynamic CTP images (Supplemental Figure 1). In total, 1024 noisy tissue TDCs were generated for each set of perfusion parameters by random sampling of Gaussian distributions with *σ* =1.5 HU. Partial volume effect, hematocrit ratio, and tissue density were neglected or set to 1.

Simulated noisy tissue TDCs were arranged in a square pattern as in previous studies^17,21^ and as illustrated in Supplemental Figure 2 by the ground truth perfusion values. Briefly, the 1024 noisy tissue curves with the same ground truth perfusion parameter were arranged in a $32\times32$ tile. Tiles were then arranged in a $7\times7$ grid according to their ground truth $T_{0}$ and MTT. MTT varied by column (longest MTT in the leftmost column, shortest MTT on the rightmost column) and $T_{0}$ varied by row (shortest on the topmost row, longest on the bottommost row). CBV was constant for each $7\times7$ grid but varied over the slice axis. CBF increased from left to right (long to short MTT) and varied according to the CBV in each slice. The described procedure generated a simulated CTP study of 8 slices and 40 time points over 80 s for each slice. The dynamic images of the digital perfusion phantom were saved as DICOM files using a rescale slope of 0.1 to maintain voxel value precision up to a tenth of a decimal.

Each CTP software (model-independent deconvolution, model-dependent deconvolution, and CTP4D) processed the digital perfusion phantom independently using the original arterial TDC and performed voxel-wise deconvolution of each tissue TDC. The software did not filter the dynamic CTP images because the injected noise was already at levels expected after filtering. To match the generation of the phantom, partial volume effect, hematocrit ratio, and tissue density were neglected or set to 1.

**
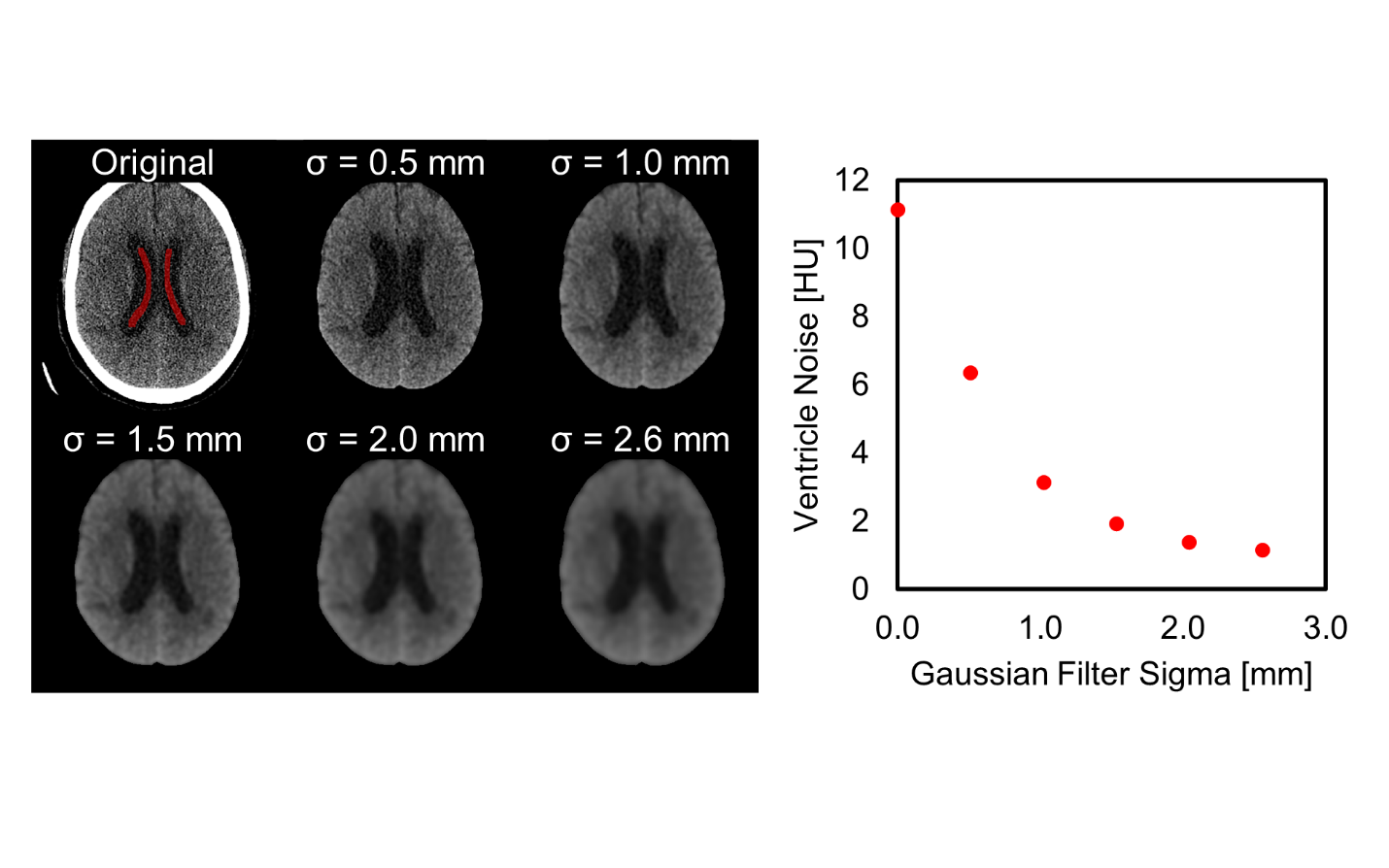
**

Supplemental Figure 1. Noise level expected in dynamic CT perfusion images after filtering with Gaussian kernels of different strengths ($\sigma$). Noise was estimated in a uniform intracranial region, i.e., the brain ventricles, as indicated by the red segmentation in the original unfiltered image. The dynamic CT perfusion study shown was acquired using a standard brain CT perfusion protocol (tube voltage: 80 kV, tube current-exposure time product: 100 mAs, slice thickness: 5 mm, 44 dynamic images over 80 s at uniform intervals). Noise was the standard deviation of delineated ventricle voxels over all 44 dynamic images. The ventricle noise at the Gaussian filter strength used in this study ($\sigma=2.4$ mm) was approximately 1.5 HU.


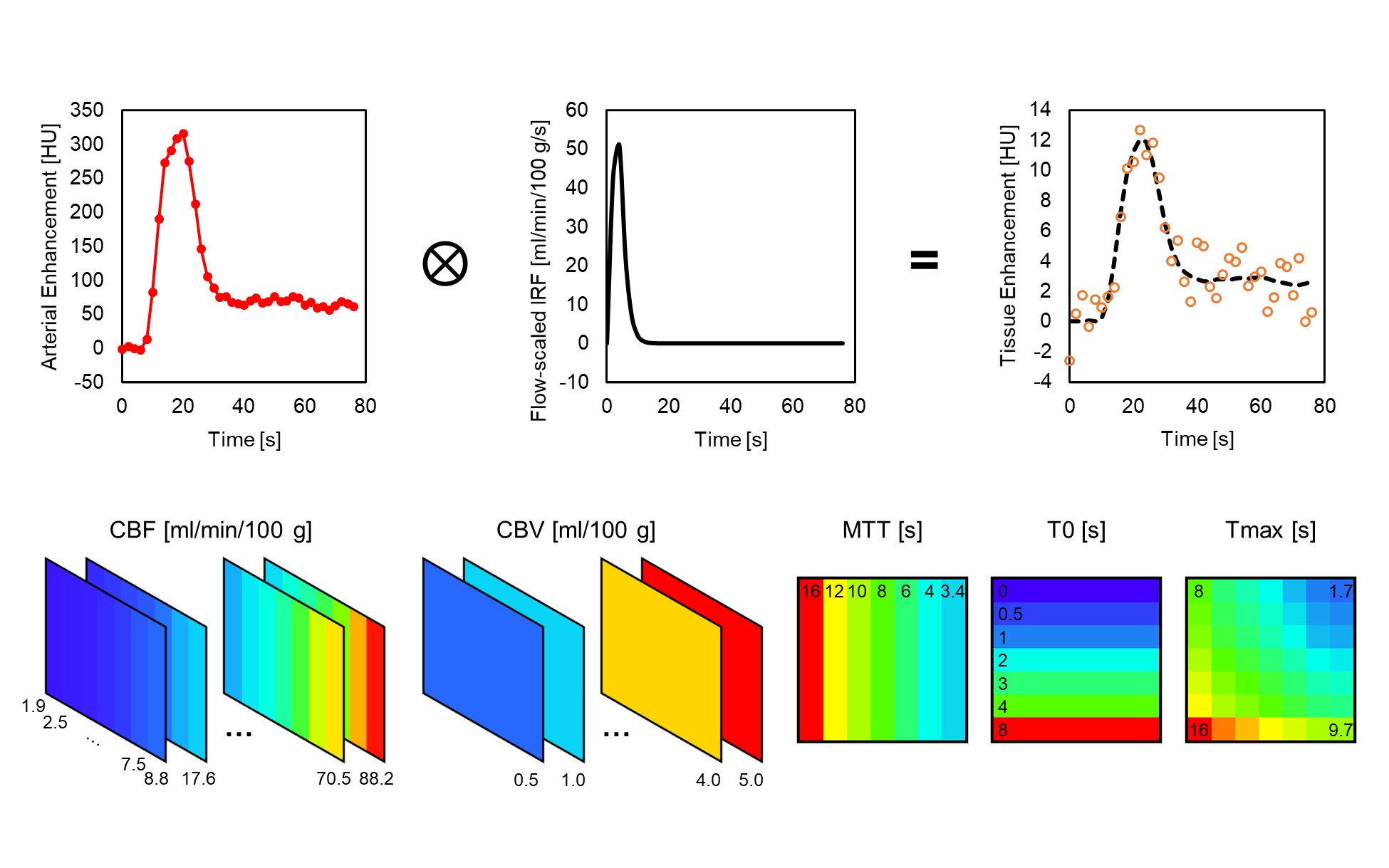


Supplemental Figure 2. Generating the digital perfusion phantom. The arterial curve (top left) was convolved ($\otimes$) with a gamma-variate flow-scaled impulse residue function (top centre) to produce a ground truth tissue curve (top right, dotted line). Gaussian noise ($\sigma=1.5$HU) was added to the simulated tissue curve. Each set of simulated perfusion parameters had a $32\times32$ tile of tissue time-density curves each with a different noise realization. Tiles were arranged in a $7\times7$ pattern as in the ground truth perfusion phantom shown in the bottom row. Cerebral blood volume (CBV) was varied by phantom slice, mean transit time (MTT) by column, delay time (T0) by row, and cerebral blood flow (CBF) by column and slice (dependent on CBV and MTT), and Tmax by row and column (dependent on T0 and MTT).

*Derivation of the Calibration Relationship*

Let Equation (9) be the linear regression of the model-independent (MI) estimate of blood flow, $F_{MI}$ against true blood flow, $F_{GT}$, in the digital perfusion phantom:

|  | $F_{MI}=\alpha_{MI} F_{GT}+\beta_{MI}$ | (9) |
| --- | --- | --- |

where $\alpha$ and $\beta$ are the slope and intercept of the linear regression, respectively. Similarly, let Equation (10) be the linear regression of the model-based (MB) estimate of blood flow, $F_{MB}$ against $F_{GT}$ in the digital perfusion phantom:

|  | $F_{MB}=\alpha_{MB} F_{GT}+\beta_{MB}$ | (10) |
| --- | --- | --- |

The calibration relationship for predicting the $F_{MB}$ that is equivalent to a $F_{MI}$ based on linear regression against $F_{GT}$ can be derived by expressing $F_{MB}$ in terms of $F_{MI}$ using Equations (9) and (10):

|  | $F_{MB}=\left( \frac{\alpha_{MB}}{\alpha_{MI}} \right)F_{MI}+\left( \frac{\alpha_{MI}\beta_{MB}-\alpha_{MB}\beta_{MI}}{\alpha_{MI}} \right)$ | (11) |
| --- | --- | --- |

Thresholds of relative blood flow, defined as blood flow normalized by that in the normal brain hemisphere, were used identify ischemic core in this study. Let $N_{GT}$, $N_{MI}$, and $N_{MB}$ be ground truth, model-independent, and model-based normal blood flow with which blood flow is normalized. $N_{MI}$ and $N_{MB}$ can be computed with $N_{GT}$ and Equations (9) and (10):

|  | $N_{MI}=\alpha_{MI} N_{GT}+\beta_{MI}$ $N_{MB}=\alpha_{MB} N_{GT}+\beta_{MB}$ | (12) |
| --- | --- | --- |

$N_{GT}=$ 50 ml/min/100 g was used in this study. The model-independent and model-based relative blood flow thresholds, $R_{MI}$ and $R_{MB}$, respectively, were then:

|  | $R_{MI}=\frac{F_{MI}}{N_{MI}}=\frac{F_{MI}}{\alpha_{MI} N_{GT}+\beta_{MI}}$ | (13) |
| --- | --- | --- |
|  | $R_{MB}=\frac{F_{MB}}{N_{MB}}=\frac{F_{MB}}{\alpha_{MB} N_{GT}+\beta_{MB}}$ | (14) |

Substituting (11) into (14) and using (13) to express the result in terms of $R_{MI}$ as shown in Equation (7) of the main text:

|  | $R_{MB}=\frac{\alpha_{MB}(\alpha_{MI} N_{GT}+\beta_{MI})R_{MI}+\alpha_{MI}\beta_{MB}-\alpha_{MB}\beta_{MI}}{\alpha_{MI}\left( \alpha_{MB} N_{GT}+\beta_{MB} \right)}$ | (7) |
| --- | --- | --- |

Equation (7) is the calibration relationship by which the equivalent model-based relative blood flow threshold to a reference model-independent relative blood flow threshold can be determined.

Using a similar methodology for $T_{max}$ would yield the following calibration equation between model-independent Tmax and model-based Tmax like that of Equation (11) and as shown in Equation (8) in the main text:

|  | $T_{max, MB}=\left( \frac{\gamma_{MB}}{\gamma_{MI}} \right)T_{max,MI}+\left( \frac{\gamma_{MI}\delta_{MB}-\gamma_{MB}\delta_{MI}}{\gamma_{MI}} \right)$ | (8) |
| --- | --- | --- |

where $\gamma$ and $\delta$ are the linear regression slope and intercept of estimated Tmax against ground truth Tmax in the digital perfusion phantom.
